# Factors affecting prevention and control of malaria among endemic areas of Gurage zone: an implication for malaria elimination in South Ethiopia, 2017

**DOI:** 10.1186/s40794-017-0060-2

**Published:** 2017-12-20

**Authors:** Tadele Girum, Gebremariam Hailemikael, Asegedech Wondimu

**Affiliations:** Department of Public health, College of Medicine and Health Sciences, Wolkite University, Wolkite, Ethiopia

**Keywords:** Malaria, Endemic area, Prevention, Practice

## Abstract

**Background:**

Globally malaria remains one of the most severe public health problems resulting in massive morbidity particularly in developing countries. Ethiopia as one of the sub-Saharan country it is highly endemic to malaria. It was noted that early detection and prompt treatment of malaria cases, selective vector control and epidemic prevention and control are the major strategies for malaria prevention and control; So far, a lot have been done and remarkable improvements were seen. However, in what extent the prevention strategy was running in the community and what factors are hindering the prevention strategy at community level was not well known in Ethiopia. Therefore this study aimed to assess measures taken to prevent malaria and associated factors among households in Gurage zone, south Ethiopia.

**Methods:**

Community based cross- sectional study was conducted in Gurage zone, southern Ethiopia**.** A total of 817 randomly selected households were included in the study. After checking for completeness the data was entered in to Epi info 7 and analyzed through SPSS (Statistical Package for Social Sciences) version 21. Descriptive summary was computed and presented by tables, graphs and figures. After checking for assumptions Bivariate analysis was run to look for the association between dependent and explanatory variables; and using variables which have *p*-value ≤0.25 binary logistic regression was fitted. Association was presented in Odds ratio with 95% confidence interval and significance determined at *P*-value less than 0.05. Goodness of fit of the final model checked by Hosmer and Lemshow test.

**Results:**

Overall 496 (62%) of households practiced good measure of malaria prevention and control. Educated households (AOR = 2.15 (95% CI [1.21–4.67]), higher wealth index (AOR = 3.3 (95% CI [2.3–6.2]), iron corrugated house owners (AOR = 2.7 (95% CI [1.7–3.5]), who received ITN from HC (AOR = 3.6 (95% CI [1.7–4.5] and involved in malaria prevention campaign AOR = 2.6, (95% CI [1.8–3.6]) were independently and significantly determined the practice of malaria prevention measures.

**Conclusion:**

The practice of malaria prevention measures were at acceptable and comparable level to other national findings and standards. Further strengthening of the program is important.

## Background

Malaria is a life-threatening disease caused by Plasmodium parasites transmitted by Anopheles mosquitoes. Plasmodium falciparum and Plasmodium vivax are the most widely distributed and pose the greatest public health threat. An estimated 3.3 billion people are at risk and 1.2 billion are considered at high risk of infection with malaria around the world [1]. In 2015, there were 214 million malaria cases and 438,000 malaria related deaths. Eighty eight percent of these malaria cases and 90% of the deaths were reported from Sub-Saharan Africa [1, 2]. Plasmodium falciparum is the most prevalent species in the African continent and is responsible for most malaria-related deaths [3].

Three quarters of Ethiopian territory is considered endemic for malaria putting 50 million people at risk for infection. Approximately 4–5 million cases of malaria and 70,000 related deaths are reported annually [4]. Malaria accounts for 30% of the overall Disability Adjusted Life Years (DALYs) lost in Ethiopia [5] making it a significant impediment to social and economic development. It is not just only a health issue but a food security and environmental issue as well [6].

The global control efforts have achieved 37% decrease in malaria incidence and 60% decline in mortality between 2000 and 2015 by applying effective prevention through indoor residual spray, use of Insecticide treated bed net (ITNs) and source reduction). Ethiopia has reduced the number of malaria cases by 50–75% [2]. This was achieved by ensuring the availability of rapid diagnostic tests and artemether/lumefantrine for detection and treatment of cases. In addition, a high coverage of ITNs distribution and spraying of households helped preventing infections and reduced malaria morbidity and mortality in the region [7]. However, malaria remains among the 10 most common causes of death in Ethiopia [8]. Possible barriers to achieve further reductions in disease burden may be associated with community involvement in prevention activities [9–11]. The extent of this involvement and its determinants are not known in the southern region of Ethiopia. Therefore, this study aimed to identify factors affecting prevention and control of malaria among communities of Gurage zone, south Ethiopia, 2016/2017.

## Methods

### Study design and settings

**Fig. 1:**
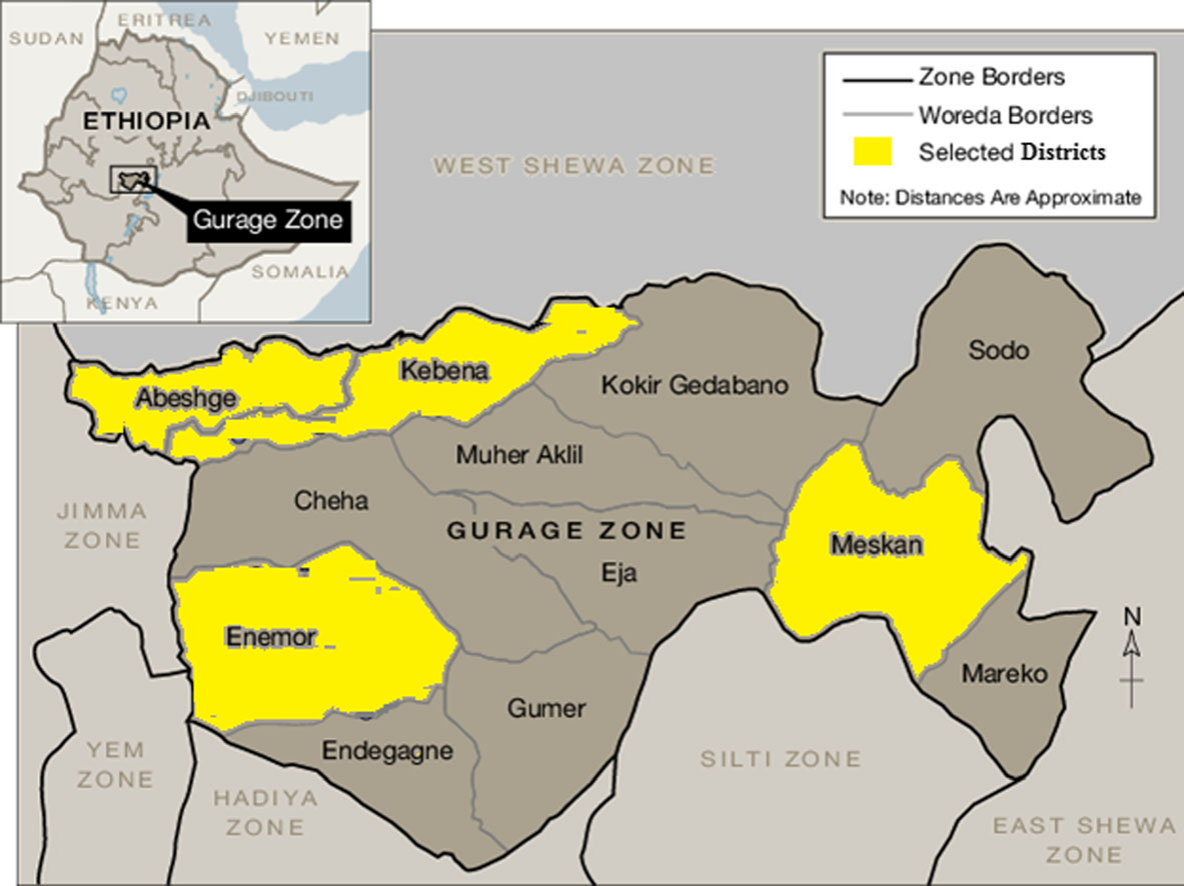
Map of Gurage zone and selected districts

This community based cross-sectional study was conducted in Gurage zone which is located in the Southern nation’s nationalities and people’s regional sate (SNNPR) of Ethiopia from January 1st to January 30th of 2017. The altitude of the Zone ranges from 1600 to 2800 m above sea level and the average annual temperature is 7–25 degree Celsius. There are seven malaria endemic districts in the zone. The zone has 13 districts, 2 town administrations, 412 rural kebeles and 32 urban kebeles. There are estimated 286,328 households in the Zone out of which 154,177 households lives in the seven endemic districts [12, 13]. Figure 1. shows map of the zone and selected districts.

### Study population and sampling technique

The Source population was all households of the seven malaria endemic districts established in the zone for more than 6 months. The Sample size was calculated based on single population proportion formula by using the formula (n = (zα/2)^2^p (1-p)/ d^2^) for estimating a single population proportion at 95% confidence interval. Three samples sizes were calculated based on key elements of the prevention strategy (use of ITN, IRS (Indoor residual spray) and knowledge of prevention) based on different reports. We used “p” for use of ITN, 0.768, “p” for use of IRS 0.59 and “p” for knowledge 0.856 [14, 15]. Then the calculated sample size includes 274, 372 and 189 respectively. The largest sample size with 10% non-response was used and multiplied by 2 for design effect; the final sample size became 817. The sample was allocated proportionally for the four randomly selected districts and systematic random sampling technique was employed in order to select individual households from each district.

### Data collection procedure and data quality control

The data collection tool was first prepared in English after reviewing related literatures and then translated to Amharic language. All the knowledge, attitude and practice assessment questions were adapted from previous researches and malaria survey questions according to local context and only a minor modification was done on each component [4, 14–20]. The questioner was pretested among 5% of households in a similar setting but outside the sampled households and found to be valid through appropriate test of Cronbach’s alpha test (>0.7). Interviewer administered interview technique was used to collect data with structured and pretested questionnaire. The mother, father or children above age of 18 were interviewed by health professionals. Whenever there were more than one eligible respondent per household the mother was preferred. Data was collected by 12 nurses after trained for a day and supervised by the investigators. The questioner sought information on: socio-demographic characteristics, information regarding malaria prevention and measured practice. Each questioner was checked for completeness by data collector and supervisors in the field (whenever it was incomplete, the questioner referred back to the house hold and completed again).

### Study variables and operational definition

The Outcome variable was practice related to prevention of malaria and the explanatory variables were: Socio-demographic characteristics (Age, Education level, Wealth status, Residence, Gender), Knowledge about malaria and attitude towards malaria.

#### Malaria prevention practice

Are practices individuals use to protect themselves against malaria. This was measured using a combination of 10 statements that used to gauge respondent’s malaria prevention and control practices. If a respondent indicate that they “always” performed a good practice, they were given a score of two points. If they indicate that they “sometimes” performed a good practice, they were given a score of one point. If on the other hand, they indicate they “never” performed a good practice, they were given a score of zero. An overall practices score was determined for each respondent by adding up the scores across the ten malaria practices questions. Then distribution of malaria prevention practices amongst respondents was scored as Good (score = > 12) and Poor (score < = 11) out of 20 points.

It asks how often they sleep in a mosquito net, other members sleep in mosquito nets, the use of mosquito repellents, they use anti-mosquito spray, they clean/cut bushes and drain stagnant water around house, they visit the health center when you fall sick, they receive visits from community health workers, they receive ITN, they participate campaign and IRS was practiced.

#### Knowledge about malaria

Awareness of respondents about basic information, signs and symptoms, transmission as well as the prevention of malaria. Overall Knowledge Score is calculated by adding up the scores for each respondent across all questions with ‘1’ for correct and ‘0’ for incorrect answers and a score of 50% and above is knowledgable.

#### Attitude towards malaria

Is feelings of respondents towards different aspects of malaria. When both positive and negative statements are scored with the right answer scoring 4 points and the wrong answer 1 point following the likert scale, an overall attitude score is determined for each respondent by adding up the scores across the 15 attitude questions. The attitude was said to be positive when the score is 53 or more.

### Data analysis

The collected data was entered into EPI INFO version 7 computer programs and exported to Statistical Package for Social Sciences (SPSS) version 21 for cleaning, recoding, categorizing and analyzing.

The distribution of the variables was described using as frequencies, means and standard deviations depending on their nature and presented in frequency tables. Some variables were dichotomized for further analysis. Bivariate analysis was conducted between outcome variable and each dependent variable. Variables with a *p*-value <.25 were considered as candidate for multiple logistic regression that was performed to test associations between categorical variables. Model fitness was checked by Hosmer and Lemeshow goodness of fit test. Later 95% CI and p-value of less than 0.05 was used to test statistical significance of the association between dependent and independent variables. Factor analysis was conducted to measure the wealth index [21] of a household.

## Results

Of the pre-selected 817 households, seven were absent through a repeated visit, three did not provide study consent seven did not have an eligible person to be interviewed. Therefore data was collected among 800 households, of whom the mean (±SD) age of the respondent was 40.5 (±11.6) year, 406 (50.8%) were female and 517(64.6%) were illiterate among the respondents. The mean (±SD) number of household members was 3.7 (±1.4) (Table 1).

**Table 1:** Socio-demographic characteristics of the study participants, Gurage zone, 2017

Characteristics	Frequency	Percent
Age		
18–24	32	4
25–34	176	22
35–44	384	48
≥ 45	208	26
Sex		
Male	394	49.2
Female	406	50.8
Marital status		
Single	80	10
Married	720	90
Level of education		
No education	517	64.6
Primary	218	27.2
Secondary	61	7.7
Tertiary	4	0.5
Occupation		
House wife/farmer	704	88
Private employer	88	11
Government employer	8	1
Wealth index		
Low	384	48
Middle	312	39
High	104	13
Family size		
≤ 2	293	36.6
3–4	430	53.8
≥ 5	77	9.6
Type of sleeping space		
Bed	168	21
Floor	632	79
Availability of ceiling		
Yes	64	8
No	736	92
Knowledge		
Yes	688	86
No	112	14
Attitude		
Positive	560	70

The majority (57.6%) of households had cottage (grass covered) traditional houses. Only 8% of the households had ceilings (structures beneath the roof where bed nets are usually hung). Nearly all (90%) of the households reported using two or more sleeping places (an area in the house used by family members to sleep that can be a bed or the floor) while the remaining had only single bed/sleeping places. Similarly majority (69%) of the houses have only one class and only 168 (21%) households have bed(a piece of furniture on or in which to lie and sleep) while others were using floor (Table 1).

Among 800 of respondents, almost all (99.5) reported that they had heard about malaria. Furthermore, 739(92.5%) of participants knew that mosquitoes play a role in transmitting the disease. About 94% of respondents correctly identified at least one symptom of malaria, fever and chills the most recalled symptoms by 593 (74.1%) and 467(58.4%) of respondents. Other symptoms identified by at least half of the respondents included feeling cold, headache, and vomiting. Most (72%) participants identified children under 5 years of age and pregnant women as the group most affected by malaria. The overall knowledge (based on 10 knowledge assessment question) about malaria among participants was (86%), while the source of information was community health workers and health professionals for 90% and 18% of participants respectively (Table 2)**.**

**Table 2:** Knowledge on malaria prevention measures in Gurage zone 2017

Characteristics	Frequency	percentage	Correct response	
			Yes (%)	NO (%)
Heard about malaria	796	99.5	99.5	0.5
Cause of malaria mentioned			74	26
Parasites	592	74		
Bacteria	128	16		
Viruses	80	10		
Signs/symptoms of malaria mentioned			94	6
Fevered	593	74.1		
chills	467	58.4		
Headache	320	40		
Joint pain	224	28		
Vomiting	176	22		
Others	39	4.9		
When mosquitoes bite mostly			89	11
Day	88	11		
Night	712	89		
Common breeding sites			88	12
Water body	704	88		
Dry area	96	12		
Common resting sites			80	20
House	640	80		
Outside house	160	20		
Mode of transmission			92.5	7.5
Mosquito bite	740	92.5		
Fly bite	36	4.5		
Drinking water	24	3		
Preventive methods mentioned (any)			99	1
ITN use	776	97		
Drainage	241	30.1		
Covering body	22	2.8		
Smoke	27	3.4		
Repellant use	141	17.6		
Close openings	22	2.8		
Advantage of mosquito nets			91	9
Prevent mosquito bite	728	91		
Attract mosquito	72	9		
Group most affected by malaria			72	28
Pregnant & children	576	72		
Other groups	224	28		
Overall knowledge				
Good knowledge	688 (86%)			
Poor knowledge	112 (14%)			

Their attitude was assessed through multiples of questions which have likert scale with four responses in terms of malaria prevention, severity, health seeking and other measurement variables. There were 70% of respondents with a positive attitude and 30% of them had a negative attitude towards malaria (Table 3).

**Table 3:** Attitude towards malaria prevention measures in Gurage zone 2017

Attitude measurement questions	Strongly Disagree N (%)	Disagree N (%)	Agree N (%)	Strongly Agree N (%)
I think that Malaria is a life-threatening disease	32(4)	40(5)	104(13)	624(78)
Malaria is a communicable disease	32(4)	64(8)	56(7)	648(81)
I think the best way to prevent myself getting Malaria is to avoid getting mosquito bites	0	0	48(6)	752(94)
I am sure that anyone can get Malaria	0	0	224(28)	576(72)
I believe sleeping under a mosquito net during the night is one way to prevent myself getting Malaria	0	24(3)	760(95)	16(2)
I am sure that self-treatment may dangers my health	0	136(17)	584(73)	80(10)
In my opinion, children and pregnant women are at higher risk of Malaria	8(1)	8(1)	704(88)	80(10)
I think that one can’t recover spontaneously from Malaria without any treatment	144(18)	104(13)	384(48)	168(21)
I think that malaria can’t transmit through contact	152(19)	136(17)	328(41)	264(33)
I might be at a greater risk of getting Malaria if I work and sleep overnight in the outside	16(2)	48(6)	624(78)	112(14)
I think that it is dangerous when Malaria medicine is not taken completely	24(3)	24(3)	648(81)	104(13)
I can buy anti-Malaria drugs from the drug shop/pharmacy to treat myself when I get Malaria	40(5)	344(43)	360(45)	56(7)
I think that I should have blood test if I have fever	40(5)	40(5)	66(528)	192(24)
I will seek for advice I get Malaria	0	0	744(93)	56(7)
In my opinion, it is very important to check for an expiry date of the drug before taking it	0	8(1)	712(89)	80(10)
Over all attitude				
Positive attitude	560 (70%)			
Negative attitude	240 (30%)			

### The use of malaria prevention methods

Among all households 776(97%) were reported that they prevent malaria through the use of ITN, while 241(30.1%) prevent through draining stagnant water and other significant proportion of respondents prevent through covering body during night time, closing openings, using of repellants and use of smoke. Majority (81.1%) of households had got ITN from the health offices in the last 1 year, for 402(50.3%) their house was sprayed with anti-mosquito and 700(87.5%) reported that they had engaged through drainage and some other environmental activity campaigns organized by the health department/facility (Table 4).

**Table 4:** Practice of malaria prevention measures in Gurage zone 2017

Practice Questions	Never N (%)	Sometimes N (%)	Always N (%)
How often do you sleep in a mosquito net?	284(35.5)	276(34.5)	240(30)
How often do other members of the household sleep in mosquito nets?	272(34)	280(35)	248(31)
How often do you use mosquito repellents in your house?	600(75)	104(13)	96(12)
How often do you use anti-mosquito spray in your house?	608(76)	112(14)	80(10)
How often your house is sprayed with anti-mosquito chemical spray (IRS) by community health workers?	392(49.7)	408(50.3)	0
How often do you clean/cut bushes around your house?	56(7)	160(20)	584(73)
How often do you clean stagnant water near your house	88(11)	624(78)	88(11)
How often do you visit the health center when you fall sick?	256(32)	160(20)	384(48)
How often do you receive visits from the community health worker?	48(6)	592(74)	160(20)
How often do you participate in malaria prevention campaigns?	16(2)	160(20)	624(78)
Over all practice			
Good practice	496(62%)		
Poor practice	304(38%)		

### Use of insecticide treated bed nets

Seven hundred thirteen (89.1%) of respondents had at least one ITN, 514(72%) of them were hanged or utilize the ITN s and the rest were not hanged the net. The net was given based on the size of the family and some of the household had more than one net. Accordingly, among all households; 321(45%) of them had more than one ITNs. Majority 406(57%) of the households reported that they don’t tucked the net under the mattress and significant number of household (7%) had one or more ITN s with tears or holes. In addition to this, 556 (78%) of respondents replied that children under 5 years age and pregnant mothers (75%) were given the priority to sleep under ITN. Forgetfulness 109(54.7) and absent of hanging material or ceiling 90(45.3%) were most mentioned reasons for not using ITN (Table 4)**.**

### Draining stagnant water

At least 700(87.5%) reported engagement of campaigns and individual practice in drainage of stagnant water, cleaning bushes and other environmental action undertaken for malaria prevention. Only 402(50.3%) houses were sprayed with anti-mosquito; none of the respondents reported painting of the house after spray was applied. When asked about treatment seeking for fever and self-medication as treatment mode for malaria, 68% of the respondents reported that they preferred to visit hospital treatment while others preferred self-medication (Table 4).

### Overall practices score

Ten practice assessment questions were prepared in order to assess the overall practice of malaria prevention and control measures among households. If a respondent indicated that they always performed a good practice in malaria prevention measures like ITN use, drainage of stagnant water, use of spray, health seeking for fever and other, they were given a score of two points, if they practice sometimes they score one and practiced not at all, given zero and the overall practice was said to be good if the score was 12 or more out of 20 points. Based on this evaluation 496 (62%) of households practiced good measure of malaria prevention and control (Table 4).

### Determinants of prevention measure (practice)

The finding shows that a strong relation was found between malaria prevention practice and marital status. Married Persons were more likely to use malaria prevention method than singles. In addition, the level of education that the respondent had attained, distribution of ITN by health professionals, community mobilization programs for malaria prevention, wealth index, type of house and presence of bed were significantly determined malaria prevention practice in the study area. By using variables which have p.value less than 0.05 in univariate analysis multivariate logistic regression was fitted and wealth index, type of house, educational status, ITN distribution and presence of community mobilization had independently determined malaria prevention practice among households.

After controlling the effect of other variables, households with the highest wealth index were 3.3 (95% CI [2.3–6.2], p.value <0.001) times more likely to practice malaria prevention and control measures than lower wealth index households. The odds of prevention practice was 2.15 (95% CI [1.21–4.67], p.value <0.001) times higher for educated families than illiterate families. Similarly the odds of malaria prevention practice among households who own iron corrugated house was 2.7 (95% CI [1.7–3.5] p.value <0.001) times higher than those who own cottage (grass covered) houses. Support from the health care system highly increased the practice of malaria prevention measures. Households who received ITN were 3.6 (95% CI [1.7–4.5] p.value <0.001) times more likely to practice prevention measure than their counter parts. Likewise initiation of community mobilization increase the odds of implementation among house holds; AOR = 2.6, (95% CI [1.8–3.6] p.value = 0.02) (Table 5).

**Table 5:** Logistic regression analysis of factors associated with malaria prevention in GZ, 2017

Characteristics	Practice of prevention		COR (95% CI)	AOR (95% CI)	*p*.value
	Good	Poor			
Marital status					
Married	458	262	1.93(1.21–3.01)	–	
Single	38	42	1	–	
Educational status					
Literate	200	83	1.8(1.32–2.45)	2.15 (1.21–4.67)	<0.001^*^
Illiterate	296	221	1	1	
Wealth index					
High	78	26	2.35(1.44–3.8)	3.3 (2.3–6.2)	<0.001^*^
Middle	203	109	1.46(1.07–2.0)	–	
Low	215	169	1	1	
Type of house					
Iron covered	224	115	1.35(1.01–1.8)	2.7 (1.7–3.5)	<0.001^*^
Cottage	272	189	1	1	
Having bed					
Yes	116	52	1.48(1.03–2.12)	–	
No	380	252	1		
Knowledge					
Good	447	241	2.38(1.6–3.57)	–	
Poor	49	63	1		
Attitude					
Positive	364	196	1.52(1.15–2.76)	–	
Negative	132	108	1		
ITN received from HC					
Yes	422	227	1.93(1.35–2.76)	3.6 (1.7–4.5)	<0.001^*^
No	74	77	1	1	
Involved in campaign					
Yes	455	245	2.67(1.74–4.1)	2.6 (1.8–3.6)	0.02*
No	41	59	1	1	
Hosmer and Lemeshow’s test			Chi-square	df	*p*.value
			14.46	8	0.07

*significant at p.value < 0.05; *GZ* Gurage Zone, *COR* crude odds ratio, *AOR* adjusted odds ratio

## Discussion

This study assessed the factors affecting the prevention and control of malaria among communities of Gurage zone, south Ethiopia; One of malaria endemic area in the region. It was found that the community is taking a lots of preventive measures integrated with the health care system. Using ITN, drainage of stagnant water, house spray/use of repellant and health care seeking for fever as designed by the government are on practice. Educational status, wealth index, type of house, presence of ITN distribution program from the health center and involvement of the community in prevention campaigns were independently and significantly determined implementation of malaria prevention measures in the community.

The study found that 713 (89.1%) of respondents had at least one ITN and 514(72%) of them were hanged or utilize the ITN. Particularly utilization was reported to be higher among women and under five children. This prevalence and ITN utilization rate as measure for prevention of malaria was comparable to other researches conducted in Ethiopia [16]. Similarly researches conducted abroad [17–19] found that ITN utilization and prevalence has increased in the last decade after prevention strategies had accelerated.

On the other hand only 50.3% households in this study used anti-mosquito spray, which is lower than other reports [16] which may be due to a program change made by the health care system from entire house to house spray to selective house spray. Therefore only villages near to water bodies and epidemic prone areas were sprayed. Among sprayed house holders none of them painted their house after the spray; such practice may be achieved through health education programs by community health workers.

In addition it was found that large proportion of households involve in drainage campaigns, drainage of water at the compound and clearing bushes which favor mosquito breeding. The overall practice of malaria prevention through different strategies was much higher than previous findings [20]. This can be explained by intense preventive measures under taking by the government through ITN distribution, availability of health care facilities and presence of community health workers, who played a major role in malaria prevention programs.

Households with the highest economic status were more likely to practice prevention measures than the lower income households. Similar to this finding, higher practice amongst households with higher wealth index has been reported previously in Uganda [20], and Ethiopia [16]. Which could be due to the fact that, individuals from high wealth index households have better information on access and capacity to buy supplementary ITN, build favorable house for living and hence are more likely to use bed nets, spray houses and drain compound. However in some researches in contrast to ours, wealth index was not associated to prevention practice [19].

The odds of prevention practice among iron corrugated house owners were higher than cottage house owner. This may be due to socio-economic difference and educational status of iron corrugated house owners which is common in semi urban areas. On the other hand the houses structural challenges, including difficulty in spreading a net over a sleeping material or a mattress, lack of a suitable structure for net hanging and disruptive sleeping arrangements that complicate bed net use in cottage houses [16]. Also favorability of the bed to tuck the net under the mattress determines ITN, use and further affects malaria prevention practice.

The malaria prevention programs implemented at the health care system also highly determines malaria prevention measure undertaking at household levels. In this regard the odds of prevention measure were higher among house holds who received ITN from health center than others who don’t get ITN at all. This could be due to the fact that availability of ITN increases the utilization and further increase measures undertaken to malaria prevention at household level. Similarly those households involved in malaria prevention campaigns were more likely to practice malaria prevention measures than those who don’t involve at campaigns.

As information is the first step for practice households lead by educated individuals were more likely to practice malaria prevention practice than illiterates; AOR = 2.6, (95% CI [1.8–3.6] p.value = 0.02). In line to our result some other researches also reported higher probability of malaria prevention measures undertaken by educated individuals than the illiterate individuals [16, 19, 20].

Some methodological problems may have encountered in this research. First it is a cross-sectional study in which risk is not measured directly. Second practice was measured from house hold report; it could be good if it was collected from observation. Third recall bias related to some variables and social desirability bias may further inflate the practice of malaria prevention measures among households.

## Conclusion and recommendation

Among all households 776(97%) were reported that they prevent malaria through the use of ITN, while 241(30.1%) prevent through draining stagnant water. Majority (81.1%) of households had got ITN from the health offices in the last 1 year, for 402(50.3%) their house was sprayed with anti-mosquito and 700(87.5%) reported that they had engaged through drainage and some other environmental activity campaigns organized by the health workers. Overall 496 (62%) of households practiced good measure of malaria prevention and control. The practice of malaria prevention measures were at acceptable and comparable level to other national findings and standards. Educational status, wealth index, type of house, presence of ITN distribution program from the health center and involvement of the community in prevention campaigns were independently and significantly determined implementation of malaria prevention measures in the community.

Therefore, further to increase the practice of malaria measures undertaken at house hold level, ITN distribution programs should be strengthened, the house to house spray program should cover larger geographical areas and the community should take active role in total community led campaign programs. Furthermore increasing academic knowledge should be considered to address those who have lack of information and those who are illiterates. More over further increase net use among all age and gender sub-groups should be considered. Researches which address the bottle neck of this research are also recommended for researchers further to recommend a better solution.

## Abbreviations

AOR: adjusted odds ratio
COR: crude odds ratio
DALYs: Disability Adjusted Life Years
GZ: Gurage Zone
IRS: indoor residual spray
ITNs: Insecticide treated bed net
SNNPR: Southern nation’s nationalities and people’s regional sate
